# A Cytokinin Analog Thidiazuron Suppresses Shoot Growth in Potted Rose Plants via the Gibberellic Acid Pathway

**DOI:** 10.3389/fpls.2021.639717

**Published:** 2021-07-15

**Authors:** Fisun G. Çelikel, Qingchun Zhang, Yanlong Zhang, Michael S. Reid, Cai-Zhong Jiang

**Affiliations:** ^1^Department of Plant Sciences, University of California, Davis, Davis, CA, United States; ^2^Department of Horticulture, Ondokuz Mayıs University, Samsun, Turkey; ^3^College of Landscape Architecture and Arts, Northwest A&F University, Xianyang, China; ^4^Crops Pathology and Genetics Research Unit, USDA-ARS, Davis, CA, United States

**Keywords:** ethylene, GA oxidase enzymes, gene expression, internode length and thickness, microscopy, miniature rose, plant height, thidiazuron

## Abstract

Application of thidiazuron (*N*-phenyl-*N*′-1,2,3-thiadiazol-5-ylurea, TDZ), a cytokinin analog, to inhibit the leaf yellowing that occurs after pinching potted rose plants, resulted in compact plants with shorter shoots and thicker internodes. Two weeks after treatment with 100 μM of TDZ, new shoots were half as long as those in control plants, and stem diameters were about 40% greater. This effect of TDZ is associated with changes in cell architecture. Although TDZ treatment stimulated ethylene production by the plants, inhibitors of ethylene biosynthesis (2-aminoethoxyvinyl glycine) or action (silver thiosulfate) did not affect the response of plants to TDZ. We found that TDZ treatment significantly suppressed the expression of bioactive gibberellic acid (GA) biosynthesis genes encoding GA3 and GA20 oxidases and slightly increased the expression of GA catabolism genes encoding GA2 oxidase. Application of GA_3_ and TDZ together resulted in normal elongation growth, although stem diameters were still somewhat thicker. Our results suggest that TDZ regulates shoot elongation and stem enlargement in potted rose plants through the modulation of bioactive GA biosynthesis.

## Introduction

Potted miniature roses (*Rosa hybrida* L.) are popular year-round potted plants, with an increase in production for special days such as Mother’s Day and Easter in the United States. However, the leaves of potted roses turn yellow and abscise after pinching (a technique that intends to stimulate branching), as well as under low light conditions after production (Zieslin et al., 1976; Tjosvold et al., 1994). In a previous studies, we used the cytokinin analog thidiazuron (TDZ) as a tool for extending the life of potted plants (Jiang et al., 2009) and preventing leaf yellowing after pinching potted miniature roses (Çelikel et al., 2019). In addition to effective prevention of leaf yellowing, we found that concentrations of TDZ higher than 80 μM inhibited shoot growth, resulting in plants with shorter and thicker stems.

Plant height control is important in maintaining the compactness and quality of potted plants during and after production (Miller, 2012). The lower light conditions in homes and offices could result in an increase in plant height and a reduction of postharvest quality. To provide compact plants, growers apply plant growth regulators, such as ethylene (released from ethephon) (Çelikel and Demir, 2019), or more commonly, inhibitors of gibberellin biosynthesis (such as flurprimidol, ancymidol, and paclobutrazol) (Miller, 2012; Demir and Çelikel, 2019), to suppress extension growth after production. Our findings that the anti-yellowing effect of TDZ was accompanied by a marked reduction of elongation growth are therefore of considerable practical interest.

The main commercial use of TDZ is as a defoliant in cotton, an activity that results from stimulation of ethylene production and accelerated abscission when the plants are sprayed with relatively low concentrations of the regulator (Suttle, 1985, 1986). Ethylene is known to reduce extension growth in many species, and thus we hypothesized that the reduction in elongation growth in miniature rose plants is due to TDZ-induced ethylene production.

## Materials and Methods

### Plant Material and Measurements

Potted miniature roses (*R. hybrida* L. cv.; Parade^®^ Rose-Apollo^®^), grown using the standard commercial procedure in 4-inch pots, were obtained from a private farm (Rocket Farms, previously Nurserymen’s Exchange Inc.) in Half Moon Bay, CA, United States during the summer months. The potted roses were pinched 5 days before shipping to University of California, Davis, CA, United States. After treatment with plant growth regulators or inhibitors of ethylene, plants were placed in a greenhouse at 21°C day/16°C night mean temperatures and natural photoperiods. Plant growth was monitored by measuring the length of the longest shoot. The length and diameter of the third and fourth internodes were measured using a digital caliper at 5, 7, 15, 21, and 25 days after treatment.

### Treatments With Plant Growth Regulators

#### Thidiazuron

The stock solution with 10 mM of TDZ was prepared by dissolving pure TDZ (Sigma, St. Louis, MO, United States) in 1 M NaOH and diluted to a proper concentration for treatments (Ferrante et al., 2002). The same diluted concentration of NaOH was used in preparing the control treatment. Potted roses were sprayed with 0 (control), 10, 20, 40, 80, and 100 μM TDZ. The highest concentration of 100 μM TDZ was used for combined treatments with ethylene inhibitors and gibberellic acid (GA_3_).

#### 2-Aminoethoxyvinyl Glycine

One day before treatment with 100 μM TDZ, potted roses were sprayed with a 0.5 mM solution of 2-aminoethoxyvinyl glycine (AVG; Sigma) to inhibit ethylene production (Saltveit, 2005). Plants were allowed to dry for 8 h before being sprayed with 100 μM TDZ.

#### Silver Thiosulfate

Silver thiosulfate (STS) concentrate was prepared as described by Reid et al. (1980). Potted rose plants were sprayed with 0.2 mM STS to inhibit ethylene action (Serek and Reid, 1993) and allowed to dry for 8 h before being sprayed with 100 μM TDZ.

#### Gibberellic Acid

Plants were sprayed with 100 μM GA_3_ (Merck) solution as described by De La Guardia and Benlloch (1980). Some plants were then transferred to the greenhouse for evaluation; replicate plants were allowed to dry for 8 h before being sprayed with TDZ.

### Microscopy

Hand-cut transverse and longitudinal sections were prepared at the middle of the third internode at 15 days after treatment with 0 (control) and 100 μM TDZ. Cell sizes (i.e., length and width) were determined from images photographed using a binocular microscope.

### Ethylene Production

The effects of different treatments on rates of ethylene production by branches excised from the plants were determined by placing them in 20 ml airtight vials containing 2 ml H_2_O. The vials were flushed with compressed air that had been passed through a column filled with Ethysorb (aluminum oxide coated with KMnO_4_, Stay Fresh Ltd., London, United Kingdom) to remove hydrocarbons. The vials were sealed for 2 h and held at 25°C, and then 3 ml of the headspace gas was injected into a Shimadzu model GC-8A gas chromatograph fitted with an aluminum oxide column and a flame ionization detector (Yin et al., 2019). The detection limit for ethylene was 5 nl/l. Ethylene production was determined in six replicate samples from each treatment.

### Semiquantitative PCR Analyses

Samples were taken from the third and fourth internodes at 7 and 15 days after spraying with 0 (control) or 100 μM TDZ. Total RNA was extracted using TRIzol Reagent (Invitrogen, Carlsbad, CA, United States) and treated with RNase-free DNase (Promega, Madison, WI, United States) to remove any contaminating genomic DNA. The first-strand cDNA was synthesized using 2 μg of total RNA, oligo d(T) primers, random hexamers, and SuperScript reverse transcriptase (Invitrogen). This cDNA was used for semiquantitative PCR analysis (Chen et al., 2004) to determine the abundance of transcripts encoding enzymes involved in GA biosynthesis and catabolism. The amplification primers for the different target genes related to bioactive GA biosynthesis and catabolism (e.g., *GA2ox*, *GA20ox-1*, *GA20ox-2*, and *GA3ox*) and for the 26 rRNA used as an internal control are shown in Supplementary Table 1.

### Statistical Analysis

The data were analyzed using one-way ANOVA with the generalized linear model procedure of JMP 10.0 software (Cary, NC, United States). Differences among treatments were analyzed using the Student’s *t*-test (*P* < 0.05). For all treatments, we used 16 replicate plants; ethylene measurements were conducted on six replicate branches per treatment. Experiments were repeated at least three times.

## Results

### Effects of TDZ Concentrations on Shoot Length in Miniature Rose Plants

**FIGURE 1:**
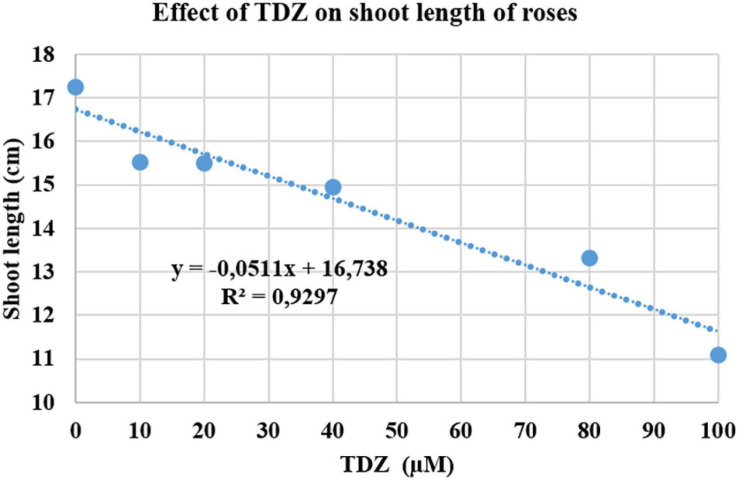
Effects of thidiazuron (TDZ) concentrations on the length of the primary shoot in miniature rose plants. Potted plants were sprayed with different concentrations of TDZ and then placed in the greenhouse for 2 weeks.

The effect of different concentrations of TDZ on the length of the primary shoot of pinched miniature roses after 2 weeks in the greenhouse is shown in Figure 1. The main shoot length was measured 2 weeks after TDZ application. The results showed a significant negative linear relationship between the length of the primary (longest) shoot and TDZ treatment concentration (Figure 1).

**FIGURE 2:**
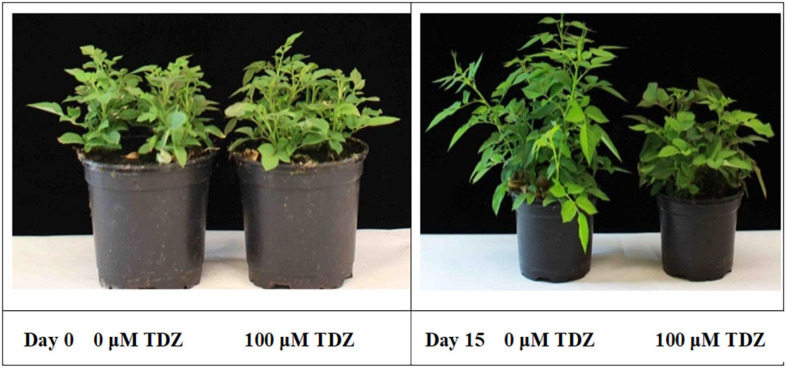
Effects of TDZ application on shoot growth of potted rose plants. Plants on the day of TDZ treatment **(left)** and 2 weeks after spraying with 0 (control) and 100 μM TDZ **(right)**.

The substantial improvement in compactness in plants treated with 100 μM TDZ, whose height was essentially unchanged after 2 weeks (Figure 2), was not accompanied by any negative effects on the plants. Therefore, we used 100 μM TDZ as the treatment in subsequent experiments.

### Effects of TDZ on Shoot Length and Thickness in Miniature Rose Plants

**FIGURE 3:**
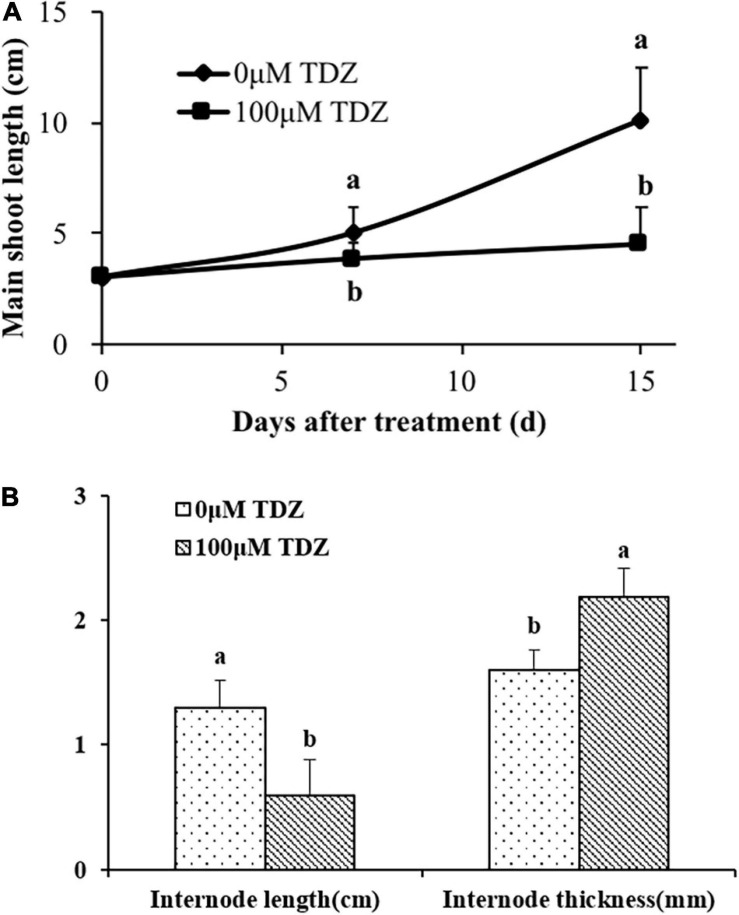
Effects of application of 100 μM TDZ on shoot length **(A)** and the third internode length and thickness **(B)** of miniature rose plants 2 weeks after treatment. Means (±SE, *n* = 16) with different letters are significantly different at *P* ≤ 0.05.

The application of 100 μM TDZ essentially prevented extension growth in potted miniature rose plants (Figure 3A). The difference from the controls was significant after 1 week, and by 2 weeks shoots on the controls were more than two times longer than those on the treated plants (Figure 3A). The internodes of the treated plants were substantially shorter and thicker than those of the control plants (Figure 3B).

### Effects of TDZ on Cell Sizes of Miniature Rose Plants

**FIGURE 4:**
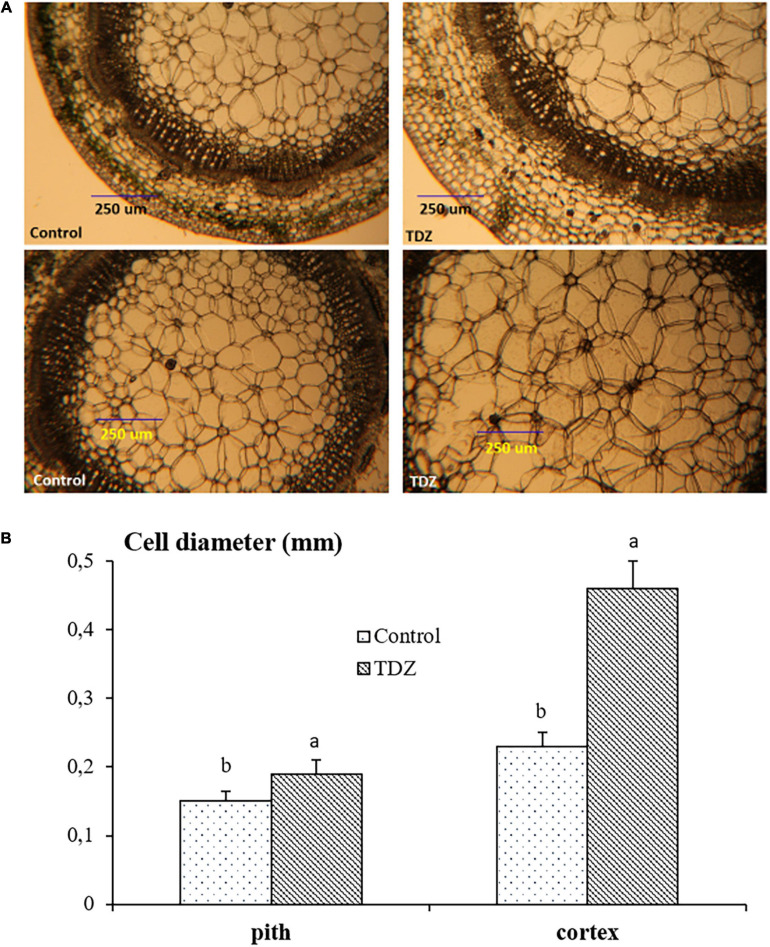
Representative hand-cut transverse sections **(A)** and mean diameters of pith and cortex cells **(B)** of third internode after 15 days of treatment with 100 μM TDZ. The data were taken around the middle sections randomly. Scale bar = 0.25 mm. Means (±SE, *n* = 16) with different letters are significantly different at *P* ≤ 0.05.

**FIGURE 5:**
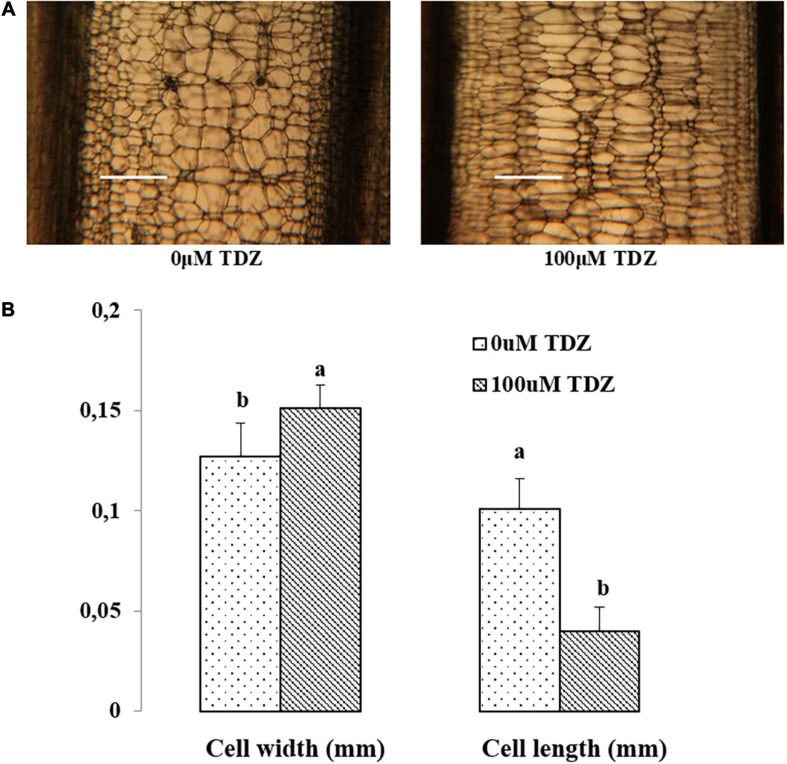
Representative hand-cut longitudinal sections **(A)** and pith cell width (mm) and length (mm) **(B)** of third internodes after 15 days of TDZ treatment. Scale bar = 0.25 mm. The data (means ± SE, *n* = 16) followed by different letters are significantly different at *P* ≤ 0.05.

Hand-cut transverse sections from the third internode were used to determine the effect of 100 μM TDZ on the diameter of cells in the cortex and the pith (Figure 4). The size of pith cells in the third internode after 15 days of treatment is shown in Figure 4B. The wider cortex and pith in the TDZ-treated plants (Figure 4A) were associated with a 25% increase in pith cell size and a 100% increase in diameter of the cortical cells (Figure 4B). Hand-cut longitudinal sections of the third internode at 15 days after treatment with 100 μM TDZ (Figure 5) showed approximately 100% radial increase in the size of the pith cells, but little change in their height (Figure 5B).

### Effects of TDZ and AVG Treatments on Ethylene Production

**FIGURE 6:**
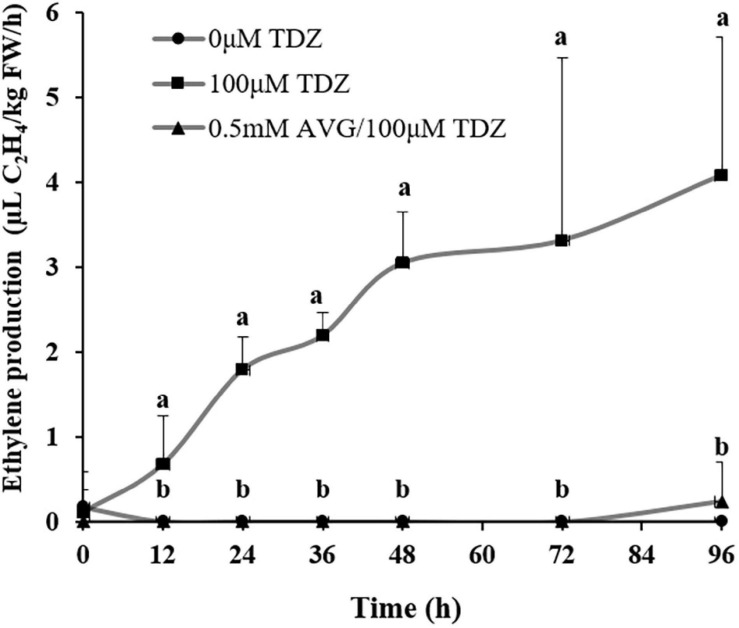
The effect of TDZ on the time course of ethylene production in shoots from control and AVG pretreated miniature rose plants. Means (±SE, *n* = 6) within days with different letters are significantly different at *P* ≤ 0.05.

Treatment with 100 μM TDZ significantly increased ethylene production of cut miniature rose shoots by 12 h (Figure 6) and production continued to increase by 96 h. This increase was suppressed by pretreatment with 0.5 mM AVG, an ethylene synthesis inhibitor (Figure 6).

### Effects of Ethylene Inhibitors and TDZ on Shoot Growth of Potted Rose Plants

**FIGURE 7:**
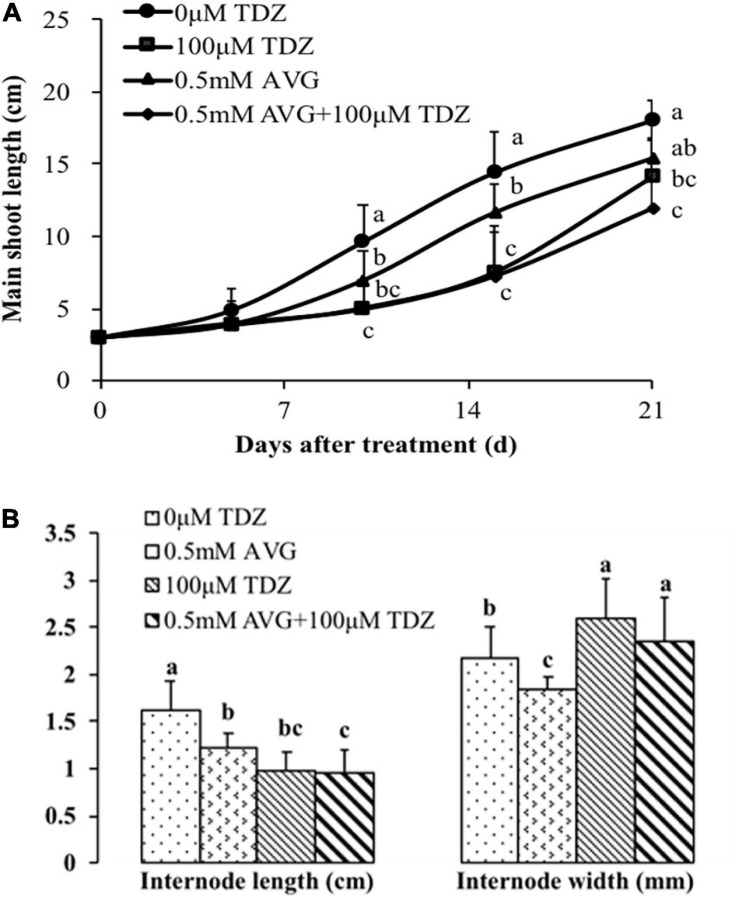
Effects of the ethylene biosynthesis inhibitor AVG at 0.5 mM and 100 μM TDZ applications alone and together on the main shoot length **(A)** and third internode length and thickness **(B)** of potted miniature rose plants. The main shoot length was measured on 5, 10, 15, and 21 days, while the data on the third internode length and thickness were taken after 3 weeks. Means (±SE, *n* = 16) within days **(A)** and length or thickness **(B)** with different letters are significantly different at *P* ≤ 0.05.

We used ethylene inhibitors to investigate the role of increased ethylene production in TDZ-treated plants on the growth response to TDZ. Pretreatment with AVG had no effect on the inhibition of shoot elongation in response to TDZ treatment (Figure 7A). Curiously, the AVG treatment alone also had a significant inhibitory effect on shoot elongation, although not as pronounced as that resulting from treatment with TDZ. These effects were mirrored in the length of the third internode (Figure 7B), but the reduction in elongation resulting from AVG treatment was not associated with increased stem diameter.

**FIGURE 8:**
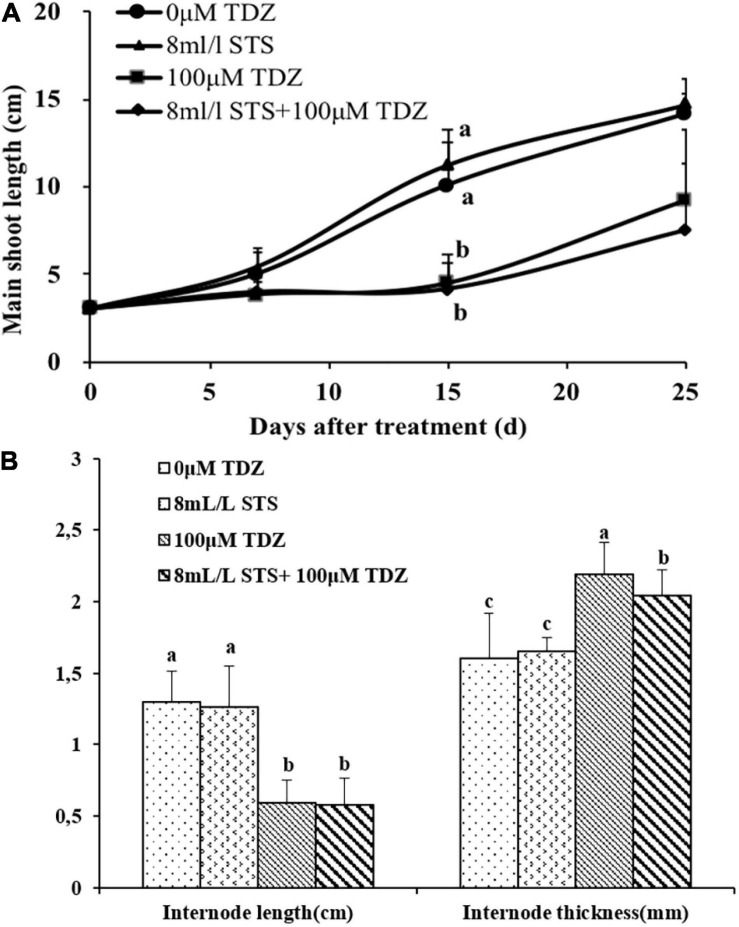
Effects of ethylene action inhibitor STS and TDZ applications alone and together on the main shoot length measured on 1, 2, and 3 weeks **(A)**, and third internode length and thickness (width) measured after 2 weeks **(B)** of potted miniature rose plants. Means (±SE, *n* = 16) within days **(A)** and length or thickness **(B)** with different letters are significantly different at *P* ≤ 0.05.

The ethylene action inhibitor STS had no effect on shoot elongation as seen in the main shoot length (Figure 8A) and third internode length (Figure 8B) in miniature rose plants. TDZ significantly inhibited shoot elongation and combined treatment with STS did not change the effect of TDZ (Figure 8), although the thickness of stems with the combined treatment was slightly less than that of stems treated along with TDZ (Figure 8B).

### Effects of TDZ on the Expression of Genes Encoding GA Oxidase Enzymes

**FIGURE 9:**
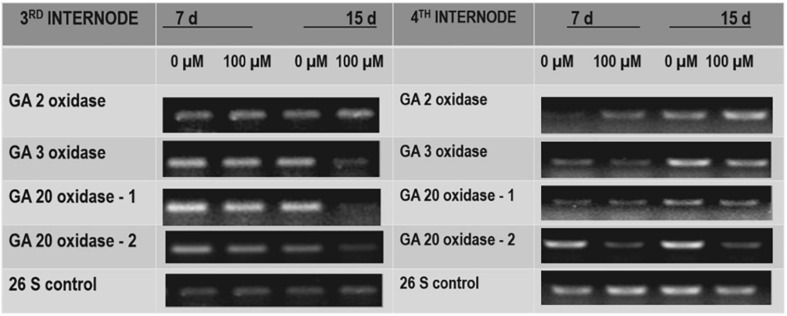
The effects of TDZ on the abundance of transcripts for gibberellic acid (GA) 2 oxidase, GA3 oxidase, and GA20 oxidase. The third and fourth internodes from the base were harvested at 7 and 15 days after TDZ treatment for RNA extraction and semiquantitative PCR analyses.

To test the effect of TDZ on GA biosynthesis in potted rose plants, we examined the effect of TDZ on the expression of genes encoding GA catabolism GA2-oxidase (*GA2ox*), which deactivates GAs, and those of some enzymes involved in bioactive GA biosynthesis (i.e., *GA3ox*, *GA20ox-1*, and *GA20ox-2*). Semiquantitative RT-PCR analysis revealed that the expression of *GA2ox* was promoted slightly by TDZ in both the third and fourth internodes (Figure 9). The expression of *GA20ox-2* was obviously suppressed by TDZ, especially in the fourth internode (Figure 9). Fifteen days after TDZ treatment, the abundance of transcripts of *GA3ox* and *GA20ox-1* was much lower, particularly in the third internode.

### The Interaction Between GA3 and TDZ on Shoot Growth of Potted Rose Plants

**FIGURE 10:**
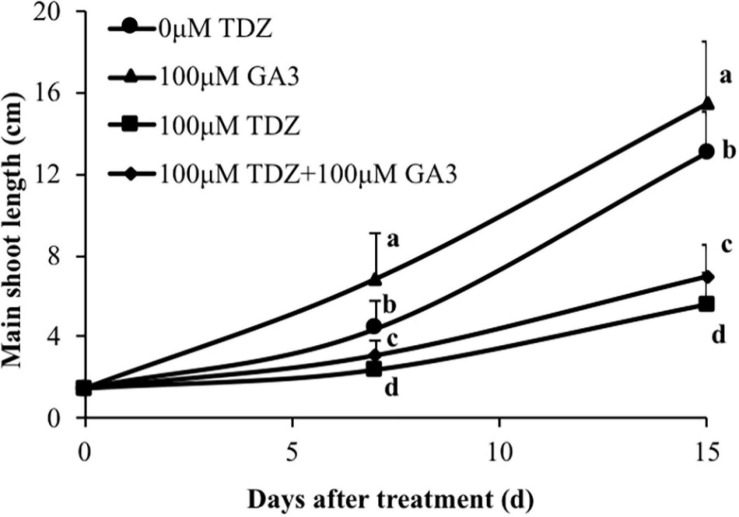
Effects of 100 μM TDZ and GA_3_ spray treatments on the main shoot length of potted miniature rose plants after 1 and 2 weeks. Means (±SE, *n* = 16) within days with different letters are significantly different at *P* ≤ 0.05.

Treatment of rose plants with 100 μM GA_3_ significantly increased extension growth in miniature rose plants (Figure 10). The inhibition of extension growth by TDZ was only modestly (although significantly) reversed by a combined treatment with the two regulators.

**FIGURE 11:**
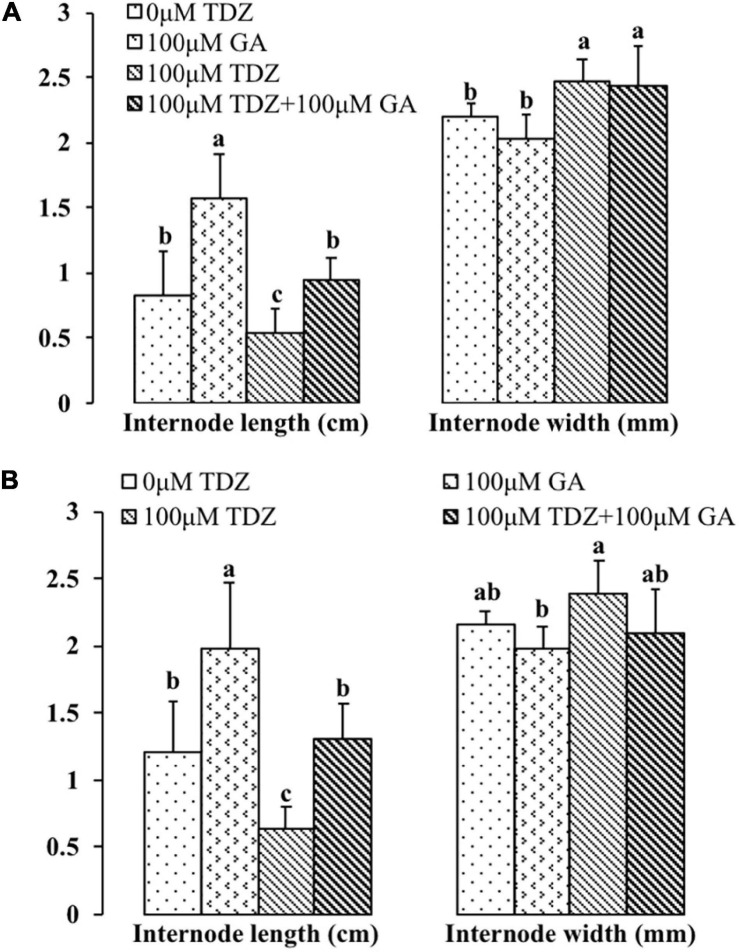
Effects of 100 μM TDZ and GA_3_ spray treatments alone and together on the third **(A)** and fourth **(B)** internode length and thickness (width) of potted miniature rose plants for 2 weeks after the treatments. Means (±SE, *n* = 16) within length or thickness with different letters are significantly different at *P* ≤ 0.05.

Gibberellic acid (GA_3_) treatment substantially increased the lengths of both the third and fourth internodes (Figure 11). In contrast to the effects of the combined treatment on the total shoot length (Figure 10), GA_3_ overcame the inhibition of elongation in the third and fourth internodes resulting from TDZ treatment. The increase in the thickness of the internodes resulting from TDZ treatment was not affected by the treatment with GA_3_ (Figure 11).

## Discussion

Controlling the plant height is important for the production and postharvest quality of potted plants (Miller, 2012). Postharvest elongation in the low light conditions of many homes and offices results in loss of compactness and visual quality. In an earlier study, we showed that TDZ reduced the leaf yellowing and abscission in potted miniature roses (Çelikel et al., 2019), an effect of this cytokinin analog that has also been shown in cut *Matthiola* (Mok et al., 1982; Ferrante et al., 2009), *Alstroemeria* (Ferrante et al., 2002), *Tulipa* and *Chrysanthemum* (Ferrante et al., 2003), and *Lupinus densiflorus* (Sankhla et al., 2005). Effects of cytokinins on leaf senescence (Wingler et al., 1998), meristem activity, and branching (e.g., Werner et al., 2001; Kyozuka, 2007; Nieminen et al., 2008) are well known. The inhibition of shoot elongation and increased stem diameter that we reported in this study is not commonly associated with cytokinin effects, although Mundhara and Rashid (2005) reported that *Linum* seedlings tissue-cultured in a medium containing 0.1 μM TDZ showed inhibition of root, hypocotyl elongation, and swelling and tightening of the cotyledons. In this study, we found a significant linear decrease in the length of the shoots of miniature rose plants as the concentration of TDZ increased up to 100 μM (Figure 1). Therefore, we used 100 μM TDZ in further experiments investigating the basis for its inhibitory effect on shoot growth in miniature rose plants, an effect with obvious commercial implications.

Plant extension growth is regulated by environmental and physiological factors, mediated by the gibberellins and ethylene. Commercially, TDZ is used to defoliate cotton leaves, a response that was shown to be the result of local stimulation of ethylene production (Suttle, 1985, 1986). Suttle (1986) concluded that the enhanced ethylene production in cotton leaves following TDZ treatment was the consequence of an increase in the formation and oxidation of the ethylene precursor 1-aminocyclopropane-1-carboxylic acid. Ethylene is known to inhibit extension growth, for example, in *Pinus sylvestris* and *Picea slauca* shoots (Little and Macdonald, 2003), sunflower hypocotyls (Pearce et al., 1991; Johnston et al., 1996), maize roots (Alarcon et al., 2009), and iris pedicels (Çelikel and van Doorn, 2015). Since TDZ treatment dramatically increased ethylene production in rose shoots (Figure 6), we hypothesized that the inhibition of extension growth in the shoots might be an effect of ethylene.

Application of AVG, which almost totally inhibited the TDZ-stimulated increase in ethylene production, did not, however, prevent the inhibition of stem growth by TDZ (Figure 7). Since AVG alone partially inhibited extension growth (suggesting a positive role for endogenous ethylene in normal growth), we tested the effect of STS, which inhibits ethylene action. STS treatment alone had no effect on the growth of the shoots (Figure 8), and the inhibitor affected neither the growth response of plants to TDZ nor the TDZ-stimulated modifications of internode architecture (Figure 8B). These data indicate that ethylene is not directly involved in the inhibitory effect of TDZ on shoot growth in miniature rose plants.

Gibberellins are important regulators of extension growth in plants, stimulating cell elongation along the longitudinal axis (Shibaoka, 1994). Stem elongation was inhibited in gibberellin-deficient *Arabidopsis* mutants (Sun and Kamiya, 1994; Chiang et al., 1995), and the gibberellin inhibitor paclobutrazol suppresses growth in a wide range of plant species by decreasing the concentrations of endogenous gibberellins (Tsegaw et al., 2005). Gibberellin inhibitors have been used during production to control the plant height after harvest (Miller, 2012) and in the production of potted bulbous flowers (Demir and Çelikel, 2019). Since the inhibition of extension growth in TDZ-treated roses did not appear to be the result of stimulated ethylene production, we investigated the effects of TDZ on expressions of genes encoding enzymes involved in gibberellin biosynthesis and degradation (catabolism). Semiquantitative RT-PCR results showed that TDZ application inhibited the expression of genes encoding the bioactive GA biosynthetic enzymes, GA20 oxidase-1, GA3 oxidase, and GA20 oxidase-2 (Figure 9). TDZ also increased the expression of genes encoding GA2 oxidase, an enzyme that inactivates endogenous bioactive GAs. Our results in miniature rose plants confirm previous data from *Arabidopsis* seedlings that cytokinin application inhibits the expression of genes encoding GA20 and GA_3_ oxidases (Brenner et al., 2005). Changes in GA20 oxidase gene expression strongly affected the stem length in potato plants (Carrera et al., 2000), and GA3-oxidase gene expression influenced GA biosynthesis and growth and development in pea (Reinecke et al., 2013). Therefore, we hypothesized that TDZ application to miniature rose plants inhibited shoot elongation by reducing the endogenous GA concentration.

We tested this hypothesis by applying GA_3_ to the control and TDZ-treated rose plants. In contrast to the effects of ethylene inhibitors, exogenous GA_3_ application combined with TDZ prevented the TDZ-mediated inhibition of shoot growth in the third and fourth internodes (Figure 11).

Cytokinins and gibberellins play important roles in the regulation of plant growth. They have antagonistic effects on shoot and root elongation, cell differentiation, and meristem activity and interact at the synthesis, catabolism, and signaling levels in the model plant *Arabidopsis* (Weiss and Ori, 2007). In the shoot apical meristem, cytokinin appears to induce the expression of *GA2ox* and promote GA deactivation for controlling the balance between cytokinin and GA (Jasinski et al., 2005). However, in the later stage of cell maturation and elongation, GA appears to play a reverse antagonistic role by inhibiting cytokinin responses for maintaining low cytokinin and high GA signals in *Arabidopsis* (Greenboim-Wainberg et al., 2005). Our results provide another example of such an antagonistic effect between cytokinin-like TDZ and GA in the control of stem maturation and elongation of a horticultural crop. The findings in this study provide an alternative approach for plant height control in maintaining the compactness and quality of horticultural plants during and after production.

The inhibition of shoot elongation by TDZ was accompanied by an increase in the stem thickness (Figure 8), and this was associated with marked changes in the cell size. The diameter of cells in the cortex doubled, and pith cell diameters increased by 25% (Figure 4B). Longitudinal sections revealed a dramatic change in the shape of the pith cells, from typically isodiametric in the controls to a flattened discoid in the TDZ-treated internodes, reflecting the inhibition of axial growth and some reallocation to radial growth (Figure 5B). Cytokinins are known to induce radial cell expansion (Shibaoka, 1994), and thus, this change is consistent with the cytokinin activity of TDZ (Nisler, 2018).

## Data Availability Statement

The original contributions presented in the study are included in the article/Supplementary Material, further inquiries can be directed to the corresponding authors.

## Author Contributions

All authors listed have made a substantial, direct and intellectual contribution to the work, and approved it for publication.

## Conflict of Interest

The authors declare that the research was conducted in the absence of any commercial or financial relationships that could be construed as a potential conflict of interest.

## Supplementary Material

Supplementary Table 1 Amplification primers for the different target genes (*GA2ox*, *GA20ox-1*, *GA20ox-2*, and *GA3ox*) and for the 26 rRNA used as an internal control.
