# Supplementary material for: A Cytokinin Analog Thidiazuron Suppresses Shoot Growth in Potted Rose Plants via the Gibberellic Acid Pathway
Source: Front Plant Sci. 2021 Jul 15;12:639717. doi: 10.3389/fpls.2021.639717 (PMC8320663; doi:10.3389/fpls.2021.639717)
Supplement: Supplementary Table 1 — Amplification primers for the different target genes (GA2ox, GA20ox-1, GA20ox-2, and GA3ox) and for the 26 rRNA used as an internal control. [file Table_1.docx]

**Table S1.** Amplification primers for the different target genes (GA2ox, GA20ox-1, GA20ox-2, and GA3ox) and the control. 26 rRNA was used as an internal control.

| Target genes and control | Forward primers | Reverse primers |
| --- | --- | --- |
| GA2ox | 5’-ATCACCATTTCTCCCAATATGC-3’ | 5’-CAAGTCATCCTCCTCTGGAATC-3’ |
| GA20ox-1 | 5’-TTTCCCTACAAACCTGAAGTCC-3’ | 5’-CTGGCGACCTACAATGGAATC-3’ |
| GA20ox-2 | 5’-GTGCTGGATTGAAGAAGAAAGG-3’ | 5’-CAAGATGAGGTTGGTGGACTG-3’ |
| GA3ox | 5’-TTATTGATTCGGAGCATCCAC-3’ | 5’-AGATCCGTTGATACAGGTACGG-3’ |
| 26 rRNA | 5’-AGCTCGTTTGATTCTGATTTCCAG-3 | 5’-GATAGGAAAGCCGACATCGAAGG-3’ |
